# A Case Report on Complicated Tuberculous Meningitis

**DOI:** 10.7759/cureus.1222

**Published:** 2017-05-04

**Authors:** Nadia Jawad, Saira Jafri, Syeda Naqvi, Syed Masroor Ahmad, Shabnam Naveed, Zeeshan Ali

**Affiliations:** 1 Chest Medicine, Jinnah Postgraduate Medical Center Karachi Pakistan; 2 Pulmonology, Jinnah Postgraduate Medical Center Karachi Pakistan; 3 Jinnah Postgraduate Medical Centre, Jinnah Sindh Medical University (SMC); 4 Medicine, Jinnah Postgrduate Medical Centre Karachi Pakistan; 5 Department of Medicine, Jinnah Postgraduate Medical Center Karachi Pakistan; 6 Jinnah Postgrduate Medical Centre, Jinnah Sindh Medical University (SMC)

**Keywords:** meningitis, tuberculous, paraplegia, motor weakness, syringomyelia, infection

## Abstract

Tuberculous meningitis (TBM) is associated with significant complications of central nervous system. It is accompanied by nonspecific and heterogeneous clinical symptoms. We focused on the significance of early diagnosis and prompt treatment. We describe a case of TBM in a 19-year-old Asian female. She had a progressive motor weakness with no sensory findings. She was started on antituberculous therapy. Her magnetic resonance imaging (MRI) contrast of dorsolumbar spine showed syringomyelia. Her culture and sensitivity for *Mycobacterium tuberculosis* (MTB) came negative. She was given a therapeutic trial of quinolones and Steroids. She had an uneventful recovery and was followed up for the past one year.

## Introduction

In the year 2015, tuberculosis (TB) infected 10.4 million people and resulted in 1.4 million deaths worldwide [1]. Prevalence in Pakistan stands at 510 per 189,000 population and mortality at 44 per 189,000 population in HIV-negative individuals [1]. Pakistan is ranked among 22 high TB burden countries. TB is the second most common fatal disease in the world. Central nervous system (CNS) TB especially tuberculous meningitis (TBM) is associated with significant morbidity and mortality [2].

Diagnosis of TBM is often delayed due to late presentation with atypical clinical features leading to high rates of morbidity and mortality. The best ways to reduce mortality and morbidity associated with TBM are the timely diagnosis, recognition of complications, and appropriate treatment [2]. Outcomes may be worsened by a low Glasgow Coma Scale (GCS), advanced stage, hydrocephalus, cranial nerve deficit, syndrome of inappropriate antidiuretic hormone (SIADH), and an abnormal electroencephalogram (EEG) at presentation [3-4]. Other neurological complications associated with TBM are stroke, seizure, hydrocephalus, vision impairment, and hearing impairment [5].

In our report, we have discussed a TBM patient with paraplegia and syringomyelia who improved on treatment. This report serves to highlight the pivotal role of timely diagnosis of unusually presenting complicated TBM in reducing morbidity.

## Case presentation

A 19-year-old married, Asian female, with a strong history of tuberculous contact, presented in a clinical set-up with a headache, backache, and mild lower limb weakness for four months. She was diagnosed as a case of TBM based on cerebrospinal fluid (CSF) detailed report. Her initial CSF report showed lymphocyte predominance, high protein, low glucose, and positive mycobacterial culture on BACTEC medium. She was started on isoniazid 250 mg, rifampin 450 mg, streptomycin 750 mg, and pyrazinamide 1000 mg once daily. After getting discharged her bilateral lower limb weakness progressed and worsened. she had become unable to even stand independently. There were associated high-grade fever, headache, and vomiting. She had no complaints of numbness, paresthesia, bowel or bladder incontinence or retention, diplopia, facial weakness, or dysphagia. Also, she did not report any trauma or fall. There was no history of cardiac, respiratory, genitourinary, gastrointestinal, or musculoskeletal abnormality. Both her parents had TB and completed treatment for it.

On admission, she was pale-looking but was vitally stable. On neurological examination, she was conscious and alert with a GCS of 15/15. Her higher mental functions and cranial nerves were intact. Signs of meningeal irritation were not present. Her sensory examination was completely normal. However, on motor examination, there was decreased bulk globally and flaccid paralysis in both lower limbs. Cerebellar signs (dysdiadochokinesia, scanning speech, intention tremors, past pointing, nystagmus) were not there. Other systemic examination findings were normal.

Initial investigations showed a normal leukocyte count. Renal function tests, electrolytes, and liver function test were within normal ranges. Erythrocyte sedimentation rate was 6 mm. Her chest x-ray (CXR) did not show any abnormality. Enzyme-linked immunosorbent assay (ELISA) for HIV came back negative. The CSF examination revealed raised CSF protein of 351 mg/dl (reference range is 15 to 60 mg/100 ml) and normal glucose of 59 mg/dl (reference range is 50 to 80 mg/100 ml). Random blood sugar (RBS) was normal (90 mg/dl) with a CSF to RBS ratio of 0.66. A lymphocytic pleocytosis was also seen. These findings except culture were quite like the prior CSF analysis based on which she was started on antituberculous therapy (ATT). The CSF microscopy, culture, and sensitivity were negative for *Mycobacterium tuberculosis* (MTB). Similarly, CSF polymerase chain reaction (PCR) could also not detect MTB DNA. Apart from this CSF oligoclonal bands were detected, indicative of intrathecal immunoglobulin G (IgG) synthesis. Magnetic resonance imaging (MRI) brain had no remarkable findings.

**Figure 1:**
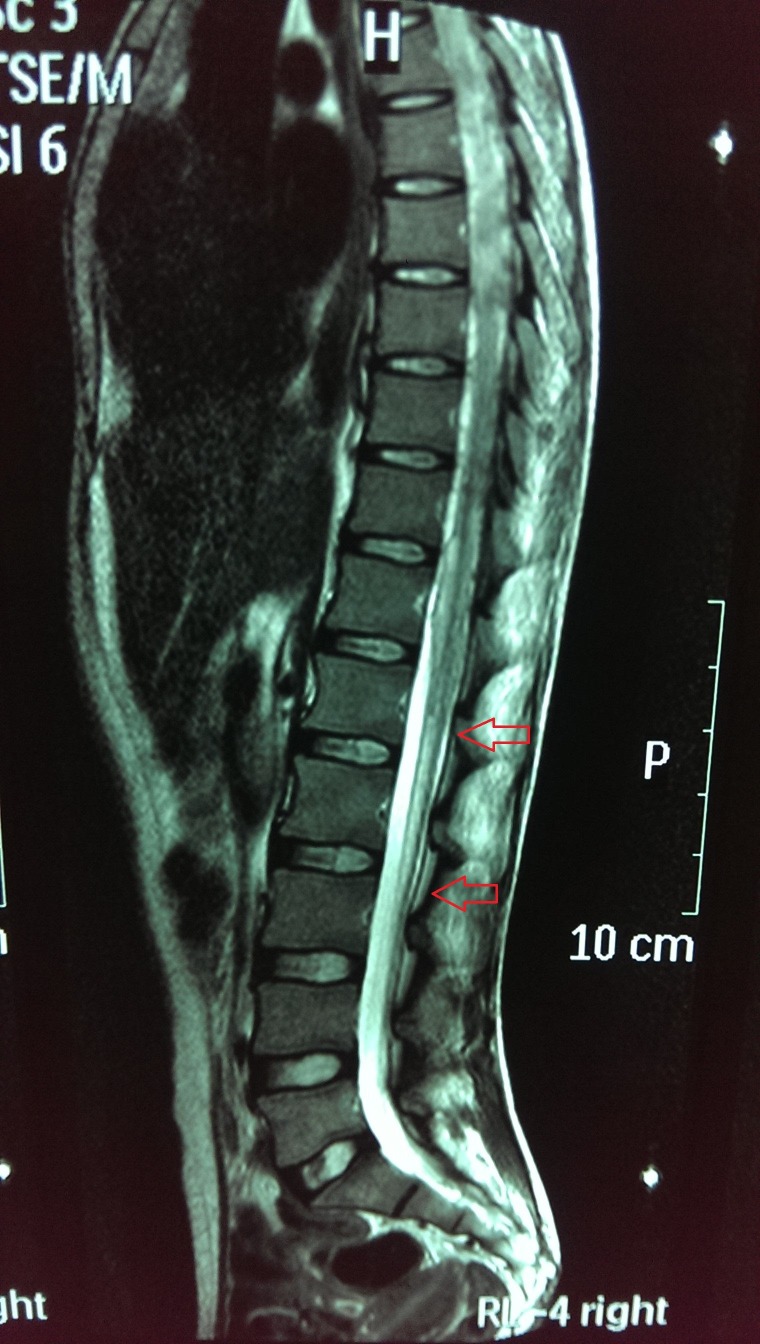
Dorsolumbar magnetic resonance imaging (MRI) spine with contrast Arrows representing syrinx formation. The cord appears low lying showng a fusiform T2W abnormal hyperintense signal area in its distal part extending from D12 to L3.

Electromyography and nerve conduction studies were suggestive of bilateral lumbosacral polyradiculopathy likely secondary to spinal arachnoiditis. MRI dorso-lumbar spine (with contrast) revealed abnormal signal intensity area seen within the spinal cord extending from the lower dorsal level up to D10 appearing iso-intense to low-intense on T1W and high-intense on T2W images showing no significant post-contrast enhancement. The cord appeared irregular in outline representing syringohydromyelia involving the long segment. There was associated clumping of peripheral nerve roots seen in the lower lumbar spine representing arachnoiditis with syrinx formation (see Figure 1).

We started her on a tapering dose of intravenous dexamethasone 0.4 mg/kg along with ciprofloxacin 750 mg twice a day while continuing oral first line ATT [6]. Her steroid dose was tapered. Over the period of one month, she showed marked improvement in power from 1/5 to 4/5 bilaterally and she could walk with support.

## Discussion

TBM most often presents with more than two to three weeks of fever, neck stiffness, and/or altered sensorium. Cranial nerve palsies and papilledema are more commonly seen in advanced stages of the disease. Patients may also present late with hemiparesis, aphasia, visual loss, seizures, and choreiform movements with the development of complications (hydrocephalus, ischemia, and abscess) [7].

The signs and symptoms of this patient suggested radiculopathy without sensory involvement. At this point, we had complicated TBM, chronic inflammatory demyelinating polyneuropathy (CIDP), multiple sclerosis (MS), and any drug toxicity as differentials. CSF analysis (with high protein, negative culture, and the presence of oligoclonal bands) and nerve conduction studies (arachnoiditis) pointed towards an inflammatory process. Most surprising was the MRI finding of syringohydromyelia in the absence of signs of cord compression. Here the diagnosis of complicated TBM became more likely.

In 2007, a case of concurrent extensive syringomyelia and intradural extramedullary tuberculoma occurring in a 27-year-old patient was described. This patient completed ATT eight months back and now developed paraparesis. She underwent surgery and was started on ATT and steroids for six months but unfortunately had no improvement [8]. By reviewing the literature it is clear that timely identification and accurate management can treat and prevent TBM complications.

The presence of syringohydromyelia makes this one of the few reported cases of concomitant TBM and syringomyelia. Syringomyelia may also be associated with isoniazid resistance alone or multidrug resistance (MDR) [9]. This became the rationale for starting a patient on ciprofloxacin together with dexamethasone. The marked improvement in motor function that was witnessed further strengthened the diagnosis. This report is meant to enlighten this presentation of TBM and to highlight the role of steroids and second line agent in marked recovery and reducing mortality [6,10].

## Conclusions

Physicians practicing in the Third World come across TB in various forms frequently. Because of the protean presentation of TB of CNS, it is often misdiagnosed and add to the mortality of this disease. While seeing a patient with motor weakness, TBM should form a strong differential among the possible diagnoses. In addition, the absence of sensory findings does not exclude the development of complications such as syringohydromyelia. Patients not responding or worsening on conventional ATT should be evaluated for MDR and coexistent syrinx formation. There is no time frame to develop neurological sequelae; it can be during treatment or even after completion of treatment. Physicians need to be vigilant in the evaluation of hearing, visual function, the appearance of the optic disc, motor function, and neurological and mental development on follow-up appointments. Prognosis of the disease depends on the duration of symptoms and management given. Steroids along with quinolones can play a decisive role in treating nervous complications of TB.

Although syringomyelia is a very rare complication of TBM but future research can be directed towards determining the relationship between the development of syringomyelia and ATT resistance.
